# Functional Magnetic Resonance Imaging Reveals Early Connectivity Changes in the Auditory and Vestibular Cortices in Idiopathic Sudden Sensorineural Hearing Loss With Vertigo: A Pilot Study

**DOI:** 10.3389/fnhum.2021.719254

**Published:** 2021-09-27

**Authors:** Qiuxia Wang, Qingguo Chen, Ping Liu, Jing Zhang, Liangqiang Zhou, Liyan Peng

**Affiliations:** ^1^Department of Radiology, Tongji Hospital, Tongji Medical College, Huazhong University of Science and Technology, Wuhan, China; ^2^Department of Otorhinolaryngology, Tongji Hospital, Tongji Medical College, Huazhong University of Science and Technology, Wuhan, China

**Keywords:** neural plasticity, idiopathic sudden sensorineural hearing loss, resting-state functional magnetic resonance imaging, regional homogeneity, vertigo

## Abstract

The underlying pathophysiology of idiopathic sudden sensorineural hearing loss (ISSNHL) with vertigo has yet to be identified. The aims of the current study were (1) to elucidate whether there are functional changes of the intrinsic brain activity in the auditory and vestibular cortices of the ISSNHL patients with vertigo using resting-state functional magnetic resonance imaging (rs-fMRI) and (2) whether the connectivity alterations are related to the clinical performance associated with ISSNHL with vertigo. Twelve ISSNHL patients with vertigo, eleven ISSNHL patients without vertigo and eleven healthy subjects were enrolled in this study. Rs-fMRI data of auditory and vestibular cortices was extracted and regional homogeneity (ReHo) and seed-based functional connectivity (FC) were evaluated; the chi-square test, the ANOVA and the Bonferroni multiple comparison tests were performed. Significantly decreased ReHo in the ipsilateral auditory cortex, as well as increased FC between the inferior parietal gyrus and the auditory cortex were found in the ISSNHL with vertigo groups. These findings contribute to a characterization of early plastic changes in ISSNHL patients with vertigo and cultivate new insights for the etiology research.

## Introduction

Idiopathic sudden sensorineural hearing loss (ISSNHL) is defined as a sensorineural hearing loss of at least 30 dB for three or more contiguous audiometric frequencies. Idiopathic sudden sensorineural hearing loss typically develops over 72 h and affects 10, 20 and 300 out of every 100,000 people in China, the United States and Germany every year (Fang et al., 1999; Michel, 2011; Stachler et al., 2012). Furthermore, vertigo develops in approximately 20% to 60% of the patients with ISSNHL (Moskowitz et al., 1984; Park et al., 2001; Rauch, 2008; Pogson et al., 2016; Chang et al., 2018). Vertigo indicated a poor prognosis for hearing recovery as the incidence of severe or profound hearing loss in ISSNHL patients with vertigo was increased (Shaia and Sheehy, 1976; Wang et al., 2009; Kim et al., 2018; Zhou et al., 2018). Chang et al. concluded that sudden hearing loss with vertigo portended greater stroke risk than sudden hearing loss or vertigo alone (Chang et al., 2018). Moreover, the burden of ISSNHL with vertigo on the patient is considerable since the impact of both the hearing loss and vertigo cannot be adequately diminished as there is a lack of effective treatments. Therefore it’s necessary to identify the underlying pathophysiology of ISSNHL with vertigo which represents a unique clinical entity dissimilar to ISSNHL without vertigo (Rauch, 2018).

Brain structural alterations have been reported in patients with auditory impairments such as unilateral hearing loss, tinnitus or deafness (Fan et al., 2015). Resting-state functional magnetic resonance imaging (rs-fMRI) has enabled the mapping of brain activity based on the blood oxygen level-dependent (BOLD) signal in ISSNHL patients. In particular, the regional homogeneity (ReHo) metric reflects the consistency of neuronal activity in a local brain region by measuring the similarity of the BOLD signal fluctuation between adjacent voxels. Historically, the ReHo metric has successfully identified biomarkers of various neurological diseases such as Alzheimer’s Disease and depression (Liu et al., 2008; Yao et al., 2009; Guo et al., 2011), and is thought to provide a pure measure of time-resolved brain connectivity patterns.

We speculated that the auditory and vestibular cortices have functional connectivity in the ISSNHL patients with vertigo. The aim of this study was to elucidate whether there are changes to the ReHo signals in the central auditory and vestibular cortices of the ISSNHL patients with vertigo and how the auditory-vestibular cortex is integrated. Another purpose was to determine whether these connectivity variations related to clinical performance changes occur during the onset of the disease.

## Materials and Methods

### Participants

This study was approved by the Institutional Review Board of the Ethics Committee of the Huazhong University of Science and Technology. Informed consent was obtained by each subject before participating in this study. Patients were enrolled between January 2017 and December 2018.

Twelve right-handed, previously untreated patients with acute unilateral ISSNHL with vertigo participated in this study. The pure tone audiometry and dizziness handicap inventory (DHI) scores were listed in Table 1. All patients met the following inclusion criteria: (1) suffering from ISSNHL for the first time; (2) unknown cause of hearing loss; (3) the level of hearing loss was at least 30 dB in at least three contiguous frequencies with no air-bone gap; (4) a history of vertiginous episodes near the onset of hearing loss; (5) the interval between the onset of vertigo and the MRI examination was ≤7 days; (6) absence of other neurological signs existed; (7) CT and MRI were performed to ensure normal ear structure and no brain lesions. The exclusion criteria included: (1) vertigo caused by benign paroxysmal positional vertigo, Meniere’s disease or acute vestibular neuritis; (2) fluctuating hearing loss; (3) inflammation of the external or middle ear; (4) a history of ear surgery; (5) spatial claustrophobia. The concomitant symptom of vertigo was defined as episodic rotational vertigo which occurred one day before/after hearing loss and lasted for several hours to several days. The onset was not related to head position and vertigo attacks did not recur after recovery.

**TABLE 1:** Demographic and clinical characteristics.

	Loss	Vertigo	*F/X* ^2^	*P*
Number (n)	11	12		
Sex (n)				
Female	7	8	0.023	0.879^†^
Male	4	4		
Age (year, mean ± SD)	41.27 ± 14.16	44.42 ± 10.57	2.146	0.550
Ear affected				
Left	6	6	0.048	0.827
Right	5	6		
Pretreatment PTA (mean ± SD)	91.2 ± 18.98 dB	97.03 ± 15.95 dB	0.877	0.362
Postreatment PTA (mean ± SD)	56.29 ± 23.61 dB	74.38 ± 22.30 dB		
DHI (mean ± SD)	-	62.67 ± 10.77		
PTA gain (n)				
No recovery	2	7	3.884	0.049^‡^
Partial recovery	8	5		
Complete recovery	1	0		

*n means number. Loss: ISSNHL without vertigo; Vertigo: ISSNHL with vertigo; PTA, pure tone audiometry; DHI, dizziness handicap inventory. p < 0.05 was considered statistically significant.*

*^†^P value was derived from Fisher’s Exact test. ‡ The comparison was between “No recovery” group and “Partial+Complete” recovery group and P value was derived from Fisher’s Exact test.*

Eleven right-handed, age, gender, and education matched patients with acute unilateral ISSNHL without vertigo were included (Table 1). The patients met the same inclusion criteria as the ISSNHL with vertigo group except the items 4 and 5.

Eleven age, gender, and education matched healthy people with normal hearing and negative otoscopic findings were included as the control group in the study. The control group had no history of auricular or neurological diseases.

### Hearing and Vestibular Testing

Pure tone audiometry testing (CONERA OB922 Audiometer, Madsen, Denmark) was performed for all participants. The pure-tone hearing thresholds at 250, 500, 1000, 2000, 4000, and 8000 Hz were recorded at the beginning and the end of the therapy. The outcome assessment was performed according to the American clinical practice guidelines.

Acoustic reflex measurements were performed by an acoustic impedance audiometer (Impedance Audiometer, Itera, Madsen, Denmark). Ipsilateral and contralateral stapes reflexes were examined at 500, 1000, 2000, and 4000 Hz. For each of these four frequencies, an acoustic stimulus of 80 dB HL was presented, and an additional 10 dB was used until a reflex curve was detected. To avoid acoustic trauma, a maximum acoustic stimulus of 110 dB was applied. The reflex curves were recorded and plotted and the latencies of the reflexes were calculated at the intersection of the baseline with the rising edge of the reflex curves.

The symptom of vertigo was assessed using the Chinese version of the dizziness handicap inventory (DHI) (Fang et al., 1999). Peripheral vestibular excitability was tested using videonystagmography with bithermal caloric irrigation (ICS CHARTR 200, Otometrics, Germany) and saccular function was assessed via the cervical vestibular-evoked myogenic potential (cVEMP) using a Medelec Synergy unit (ICS CHARTR diagnostic systems MOU-90, Otometrics, German). In all the ISSNHL patients with vertigo, the initial nystagmus examination was performed at the first visit and daily during the acute stage. Spontaneous nystagmus was checked for in an upright-seated position. Nystagmus examination lasted for 2 min.

### Resting-State Functional Magnetic Resonance Imaging Data Acquisition

fMRI data was collected using a 3.0 T MRI scanner (GE Medical Systems, Milwaukee, WI) equipped with a 32-channel head coil in the department of Radiology. The head of the subject was fixed in a head coil using rubber pads and both ears were plugged. Patients were instructed to close eyes during the functional scans. Anatomical imaging included a high-resolution three-dimensional sagittal magnetization-prepared rapid acquisition gradient echo T1-weighted sequence with the following parameters: repetition time (TR) = 5000 ms, echo time (TE) = 2960 ms, flip angle = 12 °, field of view (FOV) = 256 × 256mm^2^, matrix size = 256 × 256, slice thickness = 1 mm, no slice gap, voxel size = 1.0 × 1.0 × 1.0 mm, and slice number = 184. The resting-state functional images were acquired using a single-short gradient-echo echo-planar imaging sequence parallel to the anterior commissure-posterior commissure plane with the following parameters: TR = 2000 ms, TE = 35 ms, flip angle = 90 °, FOV = 224 × 224 mm^2^, matrix size = 64 × 64, slice thickness = 3.5 mm, no slice gap, voxel size = 3.5 × 3.5 × 3.5 mm, and slice number = 40.

### Resting-State Functional Magnetic Resonance Imaging Data Processing

The fMRI data were processed with SPM8^1^ and Data Processing & Analysis of Brain Imaging (DPABI) software.^2^ Preprocessing included: (a) discarding of the first 10 time points, (b) slice timing correction, (c) realignment and motion correction (framewise displacement, FD) (Power et al., 2013), (d) co-registration of the individual anatomical and the realigned functional volumes, (e) spatial normalization into Montreal Neurological Institute (MNI152) space through Diffeomorphic Anatomical Registration Through Exponentiated Lie Algebra (DARTEL) (Ashburner, 2018), (f) spatial smoothing with 6 mm full width half maximum (FWHM) Gaussian (this step was only performed for functional connectivity, and the ReHo was smoothed lastly), (g) reduction of confounding factors via linear regression, including the signals from the white matter and cerebrospinal fluid, and linear and quadratic trends, (h) temporal filtering (0.01–0.1 Hz) of the time series, and finally (i) motion scrubbing (Power et al., 2012; Yan et al., 2013) with a threshold of 0.5. According to the realignment parameters of fMRI run head motion, subjects were excluded from the analysis if they showed motion more than 2.0mm maximum displacement in any of the x, y, or z directions or more than 2.5° of angular motion.

### Regional Homogeneity and Seed-Based Functional Connectivity

ReHo is one of the frequently used methods to analyze image data of brain activities. An increase in ReHo means an increase in the neuronal synchrony in a specific brain region. In our experiment, the individual ReHo maps were computed using the DPABI, with the Kendall’s coefficient of concordance (KCC) algorithm and local neighborhood of 26 voxels. The ReHo maps were smoothed with a 6 mm Gaussian kernel.

A voxel-wise FC analysis of the ROI was used. The two ROI seeds were selected from the clusters that were statistically significant in the ANOVA analysis of the ReHo at the bilateral auditory regions. The Pearson correlation coefficient was obtained between all brain voxels and seed time series and was then transformed using the Fisher Z transformation to ensure a normally distributed dataset.

### Statistical Analyses

The Chi-square and Fisher’s exact tests were performed to analyse the clinical data of the ISSNHL with vertigo (Vertigo group), without vertigo groups (Loss group) and those with normal hearing (Normal group) using SPSS 22.0 software (SPSS Inc., Chicago, Illinois, United States). The results were considered significant at a threshold of *p* < 0.05.

The DPABI (edition 4.3_200401) was used for the statistical analyses. An analysis of variance (ANOVA) was used to analyze voxel-wise whole brain inter-group differences. The resultant map was corrected using a cluster-level AlphaSim algorithm (voxel *p* < 0.001 and cluster *p* < 0.05) under effective smoothing kernel estimation. This correction is equivalent to a voxel level of *p* < 0.001 and a minimum cluster size of >54 voxels. The brain regions displaying significantly different ReHo values were used to create a mask for further Bonferroni multiple comparison tests (post-hoc analyses).

The ReHo values of each group were extracted within the clusters presenting statistical significance in the ANOVA results. Correlation of the ReHo and DHI scores mentioned above was assessed using Person’s correlations. The result was considered significant at *p* < 0.05.

**FIGURE 1:**
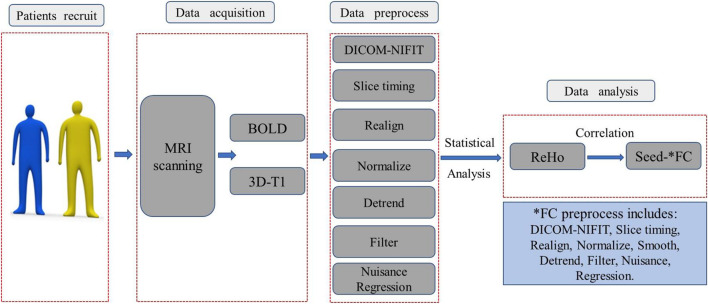
The image acquisition and statistical analysis process were summarized and depicted in this flowchart. BOLD, blood oxygen level-dependent; ReHo, regional homogeneity; DICOM-NIFIT, three-dimensional fMRI data is generally in DICOM format or NIFIT format.

The image acquisition and statistical analysis process were summarized and depicted in the flowchart in Figure 1.

## Results

### Demographic and Clinical Data

No significant difference in sex (*F* = 0.023, *p* = 0.827) or age (*X*^2^ = 2.146, *p* = 0.851) was found among the three groups. There was a significant difference between the ISSNHL without vertigo group and with vertigo group in Pure Tone Audiometry (PTA) gain for prognosis (*X*^2^ = 3.884, *P* = 0.049) (Table 1).

### Comparison of Regional Homogeneity Among Groups

**FIGURE 2:**
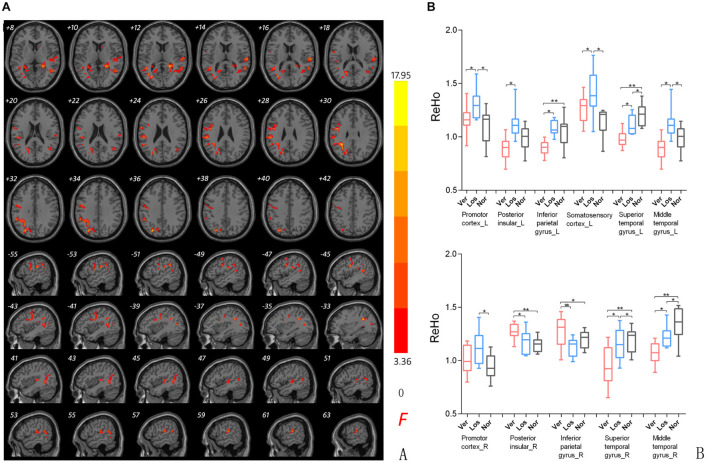
**(A)** ReHo comparison among groups. ReHo signal differences among ISSNHL with vertigo (Ver), ISSNHL without vertigo (Los), and normal hearing (Nor) groups. Slice coordinates according to Montreal Neurological Institute space are shown in the left corner of the slices, indicating the Z-axis in axial orientation and the X-axis in sagittal slices. Red denotes the regions that differed among the three groups. Differences were considered significant at a threshold of *p* < 0.001, corrected *via* Alpahsim correction. **(B)** *Post hoc* comparison of clusters showing significant differences in the three groups pairwise analysis (Ver vs. Nor, Los vs. Nor, Ver vs. Los). P*/** < 0.001.

**FIGURE 3:**
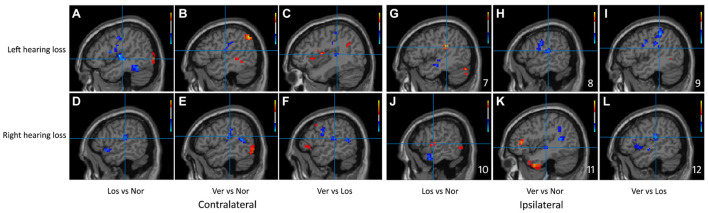
Sagittal planes of the auditory cortex were exhibited in the ReHo maps of the left and right hearing loss patients separately. The clusters of significant difference in the Bonferroni multiple comparison tests correspond to regions listed in Table 2. Slices **(A–F)** showed contralateral brain changes and **(G–L)** showed ipsilateral changes. Crosshair demonstrated the peak value of the cluster and color bars were displayed in the right corner. Ver, ISSNHL with vertigo group; Los, ISSNHL without vertigo group, and Nor, normal hearing group.

Significant differences in the ReHo values were found in both auditory and vestibular regions among the three groups, including the posterior insular, inferior parietal gyrus, superior and middle temporal regions (Figure 2); there were also differences in premotor and somatosensory cortices which are considered as vestibular related regions (Brandt et al., 1998; Frank and Greenlee, 2018). Specifically, when we focused on auditory cortex (AC) and separated the affected side, we found that the ReHo signals in the contralateral (contrary to the affected side) superior temporal gyrus were both decreased in the Vertigo and Loss groups, whereas the signals of ipsilateral (on the same side of the affected ear) AC was increased in the Loss group and decreased in the Vertigo group (Figure 3 and Table 2).

**TABLE 2:** Changes in ReHo signal of the auditory cortex by Vertigo vs. Loss vs. Normal hearing groups.

	Hemi sphere	Los VS Nor					Ver VS Nor					Ver VS Los				
		Voxel	T value	MNI coordinate			Voxel	T value	MNI coordinate			Voxel	T value	MNI coordinate		
				X	Y	Z			X	Y	Z			X	Y	Z
Left ear	L	41	3.472	–48	–33	22	53	–2.538	–50	–15	16	50	–4.101	–53	–25	25
	R	71	–3.394	48	–18	–1	46	–3.846	58	–23	13	35	–2.363	57	–22	13
Right ear	L	35	–2.420	–56	–20	19	72	–2.522	–44	–18	–4	28	–2.387	–45	–31	13
	R	63	2.342	60	–5	4	61	–3.252	53	–25	19	46	–4.962	50	–25	16

*Voxel number, T-values were obtained from the statistical parametric mapping of the ReHo (p < 0.001). The MNI coordinates reflected the center of gravity of the cluster as found in the map. Los: ISSNHL without vertigo; Ver, ISSNHL with vertigo; Nor, Normal hearing group.*

### Comparison of the Functional Connectivity Among Groups

**FIGURE 4:**
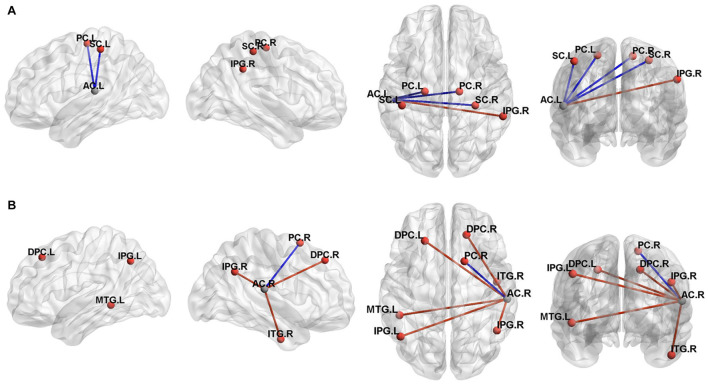
Three-dimensional rendering of the functional connectivity results (*P* < 0.001, Alphasim correction). A represented the left auditory cortex functional connectivity results and B represented the right auditory cortex results. The clusters of significant difference in the two sample *T* tests correspond to regions listed in Table 3. MNI coordinate of the ROI seed (left auditory cortex, -55/-26/12; right auditory cortex, 58/-25/16) were shown in gray ball and the positive FC connectivity areas were shown in red ball. The blue line indicated decreased connectivity and the red line indicated increased connectivity. PC, Premotor Cortex;SC, Somatosensory Cortex;IPG, Inferior Parietal Gyrus;AC, auditory cortex;MTG, Middle Temporal Gyrus;ITG, Inferior Temporal Gyrus;DPC, Dorsolateral Prefrontal Cortex;L, left;R, Right.

In the participants with the left sided hearing loss, we identified altered FC of the left AC and cerebellum with the vestibular regions and related areas such as the inferior parietal gyrus, premotor areas, somatosensory cortex, angular gyrus, V2 and posterior insular cortex. A post-hoc analysis showed a statistically significant difference in the FC between the right inferior parietal gyrus, left and right premotor cortex, left and right somatosensory cortex for the Vertigo and Loss groups (Figure 4A and Table 3).

**TABLE 3:** Brain regions with significant FC differences between ISSNHL with vertigo and without vertigo group.

Index	Brain region	ROI-1					ROI-2				
		Voxel	T value	MNI coordinate			Voxel	T value	NMI coordinate		
				X	Y	Z			X	Y	Z
1	Middle temporal gyrus (BA21_L)						115	2.678	–48	–41	–5
2	Somatosensory Cortex (BA3_L)	112	–4.962	–44	–32	56					
3	Somatosensory Cortex (BA3_R)	105	—3.312	30	–32	57					
4	Inferior parietal gyrus (BA40_L)						97	3.680	–47	–62	42
5	Inferior parietal gyrus (BA40_R)	106	3.526	58	–43	38	99	3.998	48	–56	34
6	Premotor cortex (BA6_R)	117	–3.254	14	–18	61	84	–4.874	16	12	64
7	premotor cortex (BA6_L)	101	–4.954	–21	–18	62					
8	Dorsolateral prefrontal cortex (BA9R)						116	4.095	18	38	46
9	Dorsolateral prefrontal cortex (BA9L)						90	6.655	–23	32	46
10	Inferior temporal gyrus (BA20R)						113	4.778	48	–8	–36

*Voxel number, T-values were obtained from the statistical parametric mapping of the ReHo (p < 0.001).*

*ROI-1 (MNI coordinate: -55/-26/12) and ROI-2 (MNI coordinate: 58/-25/16) served as the seeds for the FC calculation.*

*L means left brain; R means right brain; BA means Brodmann area.*

In the participants with the right sided hearing loss, we identified altered FC of the left AC and cerebellum with the premotor cortex, associative visual cortex, inferior parietal gyrus, posterior insular cortex, dorsolateral prefrontal cortex, posterior insular cortex and middle temporal gryus. A post-hoc analysis showed a statistically significant difference in the FC between the left and right inferior parietal gyrus, right middle temporal gryus and right premotor cortex for the Vertigo and Loss groups (Figure 4B and Table 3).

### Relationship Between the Dizziness Handicap Inventory Score and the Regional Homogeneity Value

**FIGURE 5:**
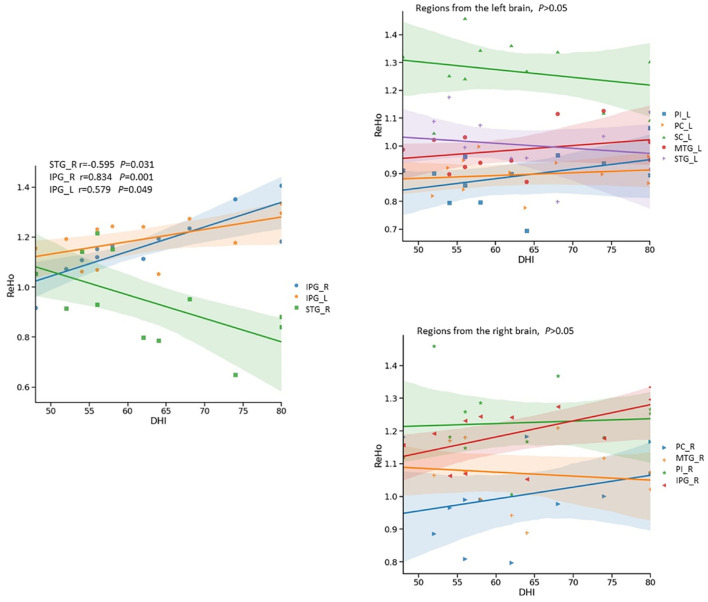
Relationship between Dizziness Handicap Index (DHI) and ReHo signals in ISSNHL with vertigo by scatter plot. The DHI showed significantly negative correlation (*r* = -0.595, *p* < 0.05) with ReHo signals from the right superior temporal gyrus, and significantly positive correlations with ReHo signals in the left (*r* = 0.579, *p* < 0.05) and right inferior parietal gyri (*r* = 0.834, *p* < 0.01). PC, Premotor Cortex; SC, Somatosensory Cortex; IPG, Inferior Parietal Gyrus; STG, Superior Temporal Gyrus; MTG, Middle Temporal Gyrus; ITG, Inferior Temporal Gyrus; PI, Posterior Insular; L, left;R, Right.

There was a significant negative correlation between the DHI scores (Table 4) and the ReHo values in the right superior temporal gyrus of the Vertigo group (*r* = -0.595, *p* = 0.031), as well as a significant positive correlation in the right inferior parietal gyrus (*r* = 0.834, *p* < 0.01) and the left inferior parietal gyrus (*r* = 0.579, *p* = 0.049) (Figure 5).

**TABLE 4:** Summary of the characteristics of ISSNHL patients with vertigo.

Patient number	Age (years)	Sex (F/M)	Affected ear	Onset time(d)	PTA (dB)		ART Ipsi/con	Vestibular test			DHI
					before	after		Spont-N	BCT	cVEMP	
1	49	F	L	4	120	85	–/85	–	+	+	56
2	61	F	R	5	85	83.3	–/85	–	–	0	58
3	38	M	L	6	105	71.6	–/85	–	+	–	64
4	57	F	L	7	88.3	71.6	–/90	R	+	+	54
5	42	F	R	2	90	90	–/85	–	+	–	48
6	31	F	L	6	120	63	–/–	L	N	+	80
7	52	M	R	5	94	64	–/80	–	–	–	58
8	45	M	R	4	75	25	–/80	–	–	+	80
9	40	F	R	7	120	120	–/85	L	+	0	74
10	25	F	R	1	92	80	–/85	–	–	–	62
11	53	M	L	5	78	60	–/80	–	N	–	52
12	40	F	L	2	97	79	–/85	–	+	+	56

*PTA, pure tone audiometry; ISSNHL, Idiopathic sudden sensorineural hearing loss; BCT, bithermal caloric test; cVEMP, cervical vestibular evoked myogenic potential; DHI, dizziness handicap inventory; “+”, abnormal of waveforms or results; “–”, normal waveforms or results; 0, no response; N, Test was not finished and terminated. ART, Acoustic Reflex Thresholds (ART) was a 3 frequency average (500, 1k, and 2k Hz) of the sensation level in dB HL; ipsi, ipsilesion; con, contralesion; “–”, absent. Spon-N, spontaneous nystamus; “–”, absent; R, right ear; L, left ear.*

## Discussion

To our best knowledge, this study firstly identified the altered activities in the auditory and vestibular cortices of the ISSNHL patients with vertigo by rs-fMRI. The major findings of this study demonstrated that: (1) the auditory and vestibular cortices both exhibited altered local activities; (2) In comparison to the normal hearing, the ReHo signals of the ipsilateral AC were increased in the ISSNHL without vertigo and decreased in the ISSNHL with vertigo; (3) There was a negative association between the DHI scores and the ReHo values in the inferior parietal gyrus. In sum, these results demonstrated that the ISSNHL patients with vertigo exhibited different intrinsic brain activity patterns compared to the ISSNHL patients without vertigo and healthy controls.

### The Auditory and Vestibular Cortices Showed Functional Changes

The presence of vertigo in some but not all ISSNHL patients suggests that the brain areas involved in the additional dizziness may demonstrate a distinctive pattern of activity. From our results, we observed the ReHo signal changes in the auditory and vestibular cortices, such as the superior and middle temporal cortices, posterior insular cortex, inferior parietal gyrus (Figure 2). Furthermore, when comparing the ISSNHL without vertigo and the vestibular neuritis, the confirmed hypothesis was obtained: in the ISSNHL patients without vertigo, the activity of the contralateral AC was decreased, and the vestibular cortex was normal (Micarelli et al., 2017); in the vestibular neuritis patients (Bense et al., 2004), the activity of the right vestibular cortices was changed (increased or decreased) and the activity of the AC was normal; in the ISSNHL patients with vertigo, the activities of the AC and left vestibular cortices were decreased and the right parieto-insular vestibular cortex and inferior parietal cortex were increased (Figure 1).

### The Activity of the Ipsilateral Auditory Cortex Was Decreased in Idiopathic Sudden Sensorineural Hearing Loss With Vertigo

The auditory projections from the inner ear to the AC cause contralateral activation of the brain in response to sound. Such a cross-projection is useful in binaural facilitation of hearing and localization of sounds (Moore, 1991). However, when unilateral hearing is deprived, the corresponding AC activities may change in response.

In the contralateral AC of the unilateral ISSNHL patients (with or without vertigo, Figure 2), we observed a reduction of the ReHo values compared to the normal hearing. The underlying mechanism of this contralateral deactivation could be related to the misrepresentation of the sound intensity at the cortical level (Musiek et al., 2013). The perception of loudness or intensity at the cortical level is affected by the interaction between the excitatory and inhibitory neurons (Phillips et al., 1994). If deprived sound input activates fewer excitatory fibers, the activities of excitatory and inhibitory neurons can become unbalanced and, as a result, the neuronal response in one hemisphere will be reduced compared to the other (Fan et al., 2015). This finding is consistent with the previous studies in patients with chronic deafness or tinnitus reporting reduced blood flow in the AC (Lanting et al., 2009; Okuda et al., 2013; Micarelli et al., 2017). However, some recent studies reported no significant difference in the ReHo value between the ISSNHL patients and the healthy controls in any brain region. The various neuroimaging methods employed and the participants’ heterogeneity may have contributed to the inconsistent results (Cai et al., 2020; Chen et al., 2020).

We observed a different effect in the activity of the ipsilateral AC when vertigo was present. The ipsilateral AC showed the restrained status after vertigo happened: the ReHo signals of the ipsilateral AC were increased in the ISSNHL group without vertigo, whereas they were decreased in the ISSNHL with vertigo (Figure 3). In two magnetoencephalography reports on ISSNHL, the activity of the ipsilateral AC was observed and elucidated. Morita and Li reported a stronger N100 evoked response in the ipsilateral compared to the contralateral hemisphere in response to ear stimulation during the acute phase of hearing loss (Li et al., 2006; Morita et al., 2007). The authors hypothesized that a cochlear lesion might induce a bilateral effect through retrocochlear crossing fibers that may influence the function of the auditory pathway associated with the ipsilateral healthy ear. This pattern of the reduced contralateral and increased ipsilateral AC activities in ISSNHL without vertigo may reflect the compensatory mechanism of the brain after unilateral hearing deprivation. However, how to explain the decreased activity of the ipsilateral AC in the ISSNHL patients with vertigo and what is the mechanism of this reduction? Did the activities of vestibular cortices lead to this reduction?

### The Functional Connectivity of the Auditory-Vestibular Cortices in the Idiopathic Sudden Sensorineural Hearing Loss With Vertigo

Our research demonstrated that the inferior parietal gyrus and the AC displayed functional connectivity in the ISSNHL patients with vertigo (Figures 4A,B). It is known that the inferior parietal gyrus belongs to the multisensory area predominantly in the temporo-insular and temporo-parietal cortex of the human brain. These multisensory areas are involved in the processing of vestibular information and have been delineated during the last 7 years by functional imaging studies in humans (Bremmer et al., 2001; Fasold et al., 2002; Emri et al., 2003; Stephan et al., 2005).

In contrast to the integration of visual-vestibular perception, less is known about the neural mechanism that mediates the integration of vestibular and auditory processing. An early human functional study revealed a consistent focal activation in the superior temporal region during vestibular activation (Friberg et al., 1985). A vestibular galvanic study demonstrated activations in the superior temporal gyrus and as well in the middle temporal gyrus (Bense et al., 2001). These results depicted a functional connectivity between the vestibular cortex and the auditory cortex in the human brain. Our findings also revealed that the inferior parietal cortex and the AC exhibited functional connectivity in the ISSNHL patients with vertigo (Figures 4A,B). A mechanism similar to the inhibitory visual-vestibular cortex interaction (Brandt and Dieterich, 1998; Della-Justina et al., 2015; Frank et al., 2020) may explain our experimental findings in the auditory-vestibular cortex of the patients. That means the dysfunction of the inferior parietal cortex perturbs the activity of the temporal cortex and worsens the auditory function. Confirmation of this theory and the mechanism or interaction must be examined in further experiments.

### Correlation Analysis

A positive correlation was found between the DHI scores and the inferior parietal gyrus activity (Figure 5). In fact, activation of inferior parietal gyrus has been seen during caloric vestibular stimulation in fMRI and PET studies (Dieterich and Brandt, 2008; Helmchen et al., 2014). Brodmann Area 40 belongs to a multisensory area in the inferior parietal lobe, part of with strong projections the temporal lobe. Monkey studies revealed two vestibular areas in the parietal lobe, named the visual temporal sylvian area and area 7b (Faugier-Grimaud and Ventre, 1989; Guldin and Grusser, 1998). The location of these regions is comparable to parts of our activation area in inferior parietal gyrus. However, these findings do not imply that the observed fMRI changes are a contributing factor to the vertigo symptom. Our results are based on an observational study focusing on the central correlates of symptoms with a peripheral origin. The mechanisms involved in the observed changes to the central auditory areas, that occur in those that develop vertigo, need further investigation.

### Limitations

Despite the promising results, there were certain limitations in our study. The small sample size of this research may cause type II statistical errors. Acute otoneurological patients, due to their very severe symptoms, often refused or delayed the MRI examination due to discomfort of lying still. This issue had made the recruitment of an adequate number of subjects difficult in functional neuroimaging studies, including ours (Micarelli et al., 2017).

Secondly, our study did not touch upon the pathogenesis of peripheral vestibular organs in this disease. The occurrence of vertigo was an integrated response of the whole vestibular pathway and it is becoming increasingly suggested that peripheral vestibular organs play a role in vertigo symptoms in the patients with sudden deafness. An overall vestibular pathway investigation should be carried out to fully uncover the pathogenesis of vertigo during sudden deafness.

## Conclusion

Our research provides the first evidence for the altered activity in the AC and vestibular cortical areas of the ISSNHL patients with vertigo by rs-fMRI. The decreased activity of the ipsilateral AC in ISSNHL patients with vertigo was our main finding. We also found that ReHo value of the inferior parietal cortex was related to the DHI scores of the patient and this area demonstrated positive functional connectivity with the AC. The auditory and vestibular cortices demonstrate functional connectivity in the ISSNHL patients with vertigo. These findings contribute to the identification of neural plasticity in ISSNHL patients with vertigo and warrant further investigation to understand the mechanisms involved in the generation of vertigo.

## Data Availability Statement

The original contributions presented in the study are included in the article/Supplementary Material, further inquiries can be directed to the corresponding authors.

## Ethics Statement

The studies involving human participants were reviewed and approved by the Institutional Review Board of the Ethics Committee of the Huazhong University of Science and Technology. The patients/participants provided their written informed consent to participate in this study.

## Author Contributions

LP, QW, and LZ developed the research ideas. LP and LZ developed hypotheses, conducted analyses, interpreted data, and drafted the manuscript. PL coded, analyzed data, revised drafts of the manuscript, and designed the data collection protocol. PL and JZ revised drafts of the manuscript, contributed in discussing analyses, and provided critical revisions of the manuscript. All authors approved the submitted version.

## Conflict of Interest

The authors declare that the research was conducted in the absence of any commercial or financial relationships that could be construed as a potential conflict of interest.

## Publisher’s Note

All claims expressed in this article are solely those of the authors and do not necessarily represent those of their affiliated organizations, or those of the publisher, the editors and the reviewers. Any product that may be evaluated in this article, or claim that may be made by its manufacturer, is not guaranteed or endorsed by the publisher.

## Footnotes

1
https://www.fil.ion.ucl.ac.uk/spm

2
http://rfmri.org/dpabi

## Abbreviations

BA: Brodmann area
BOLD: blood oxygen level-dependent
cVEMP: cervical vestibular-evoked myogenic potentials
DHI: dizziness handicap inventory
FC: functional connectivity
ISSNHL: idiopathic sudden sensorineural hearing loss
MATLAB: matrix laboratory platform
MNI: Montréal neurological institute
ReHo: regional homogeneity
ROI: region of interest
rs-fMRI: resting-state functional magnetic resonance imaging
AC: auditory cortex.

## References

[B1] Ashburner J. (2018). A fast diffeomorphic image registration algorithm. *Neuroimage* 38 95–113. 10.1016/j.neuroimage.2007.07.007 17761438

[B2] Bense S. Bartenstein P. Lochmann M. Schlindwein P. Brandt T. Dieterich M. (2004). Metabolic changes in vestibular and visual cortices in acute vestibular neuritis. *Ann. Neurol.* 56 624–630. 10.1002/ana.20244 15449325

[B3] Bense S. Stephan T. Yousry T. A. Brandt T. Dieterich M. (2001). Multisensory cortical signal increases and decreases during vestibular galvanic stimulation (fMRI). *J. Neurophysiol.* 85 886–899. 10.1152/jn.2001.85.2.886 11160520

[B4] Brandt T. Dieterich M. (1998). The vestibular cortex. Its locations, functions, and disorders. *Ann. N. Y. Acad. Sci.* 871 293–312. 10.1111/j.1749-6632.1999.tb09193.x 10372080

[B5] Brandt T. Bartenstein P. Janek A. Dieterich M. (1998). Reciprocal inhibitory visual–vestibular interaction. Visual motion stimulation deactivates the parieto-insular vestibular cortex. *Brain* 121 (Pt 9) 1749–1758. 10.1093/brain/121.9.1749 9762962

[B6] Bremmer F. Schlack A. Shah N. J. Zafiris O. Kubischik M. Hoffmann K. (2001). Polymodal motion processing in posterior parietal and premotor cortex: a human fMRI study strongly implies equivalencies between humans and monkeys. *Neuron* 29 287–296. 10.1016/s0896-6273(01)00198-2 11182099

[B7] Cai Y. Xie M. Su Y. Tong Z. Wu X. Xu W. (2020). Aberrant functional and causal connectivity in acute tinnitus with sensorineural hearing loss. *Front. Neurosci.* 14:592. 10.3389/fnins.2020.00592 32714128 PMC7340148

[B8] Chang T. P. Wang Z. Winnick A. A. Chuang H. Y. Urrutia V. C. Carey J. P. (2018). Sudden hearing loss with vertigo portends greater stroke risk than sudden hearing loss or vertigo alone. *J. Stroke. Cerebrovasc. Dis.* 27 472–478. 10.1016/j.jstrokecerebrovasdis.2017.09.033 29102540 PMC6049697

[B9] Chen J. Hu B. Qin P. Gao W. Liu C. Zi D. (2020). Altered brain activity and functional connectivity in unilateral sudden sensorineural hearing loss. *Neural Plast.* 2020:9460364. 10.1155/2020/9460364 PMC7527900 33029130

[B10] Della-Justina H. M. Gamba H. R. Lukasova K. Nucci-da-Silva M. P. Winkler A. M. Amaro E. Jr. (2015). Interaction of brain areas of visual and vestibular simultaneous activity with fMRI. *Exp. Brain Res.* 233 237–252. 10.1007/s00221-014-4107-6 25300959

[B11] Dieterich M. Brandt T. (2008). Functional brain imaging of peripheral and central vestibular disorders. *Brain* 131(Pt 10) 2538–2552. 10.1093/brain/awn042 18515323

[B12] Emri M. Kisely M. Lengyel Z. Balkay L. Marian T. Miko L. (2003). Cortical projection of peripheral vestibular signaling. *J. Neurophysiol.* 89 2639–2646. 10.1152/jn.00599.2002 12740408

[B13] Fan W. Zhang W. Li J. Zhao X. Mella G. Lei P. (2015). Altered contralateral auditory cortical morphology in unilateral sudden sensorineural hearing loss. *Otol. Neurotol.* 36 1622–1627. 10.1097/mao.0000000000000892 26595717 PMC4658668

[B14] Fang J. Q. Hao Y. T. Li C. X. (1999). Reliability and validity for Chinese version of WHO quality of lifet scale. *Chin. Ment. Health. J.* 13 203–206.

[B15] Fasold O. von Brevern M. Kuhberg M. Ploner C. J. Villringer A. Lempert T. (2002). Human vestibular cortex as identified with caloric stimulation in functional magnetic resonance imaging. *NeuroImage* 17 1384–1393. 10.1006/nimg.2002.1241 12414278

[B16] Faugier-Grimaud S. Ventre J. (1989). Anatomic connections of inferior parietal cortex (area 7) with subcortical structures related to vestibulo-ocular function in a monkey (*Macaca fascicularis*). *J. Comp. Neurol.* 280 1–14. 10.1002/cne.902800102 2465325

[B17] Frank S. M. Greenlee M. W. (2018). The parieto-insular vestibular cortex in humans: more than a single area? *J. Neurophysiol.* 120 1438–1450. 10.1152/jn.00907.2017 29995604

[B18] Frank S. M. Pawellek M. Forster L. Langguth B. Schecklmann M. Greenlee M. W. (2020). Attention networks in the parietooccipital cortex modulate activity of the human vestibular cortex during attentive visual processing. *J. Neurosci.* 40 1110–1119. 10.1523/jneurosci.1952-19.2019 31818978 PMC6989006

[B19] Friberg L. Olsen T. S. Roland P. E. Paulson O. B. Lassen N. A. (1985). Focal increase of blood flow in the cerebral cortex ofman during vestibular stimulation. *Brain* 108 609–623. 10.1093/brain/108.3.609 3876134

[B20] Guldin W. O. Grusser O. J. (1998). Is there a vestibular cortex? *Trends Neurosci.* 21 254–259. 9641538 10.1016/s0166-2236(97)01211-3

[B21] Guo W. B. Sun X. L. Liu L. Xu Q. Wu R. R. Liu Z. N. (2011). Disrupted regional homogeneity in treatment-resistant depression: a resting-state fMRI study. *Prog. Neuropsychopharmacol. Biol. Psychiatry* 35 1297–1302. 10.1016/j.pnpbp.2011.02.006 21338650

[B22] Helmchen C. Ye Z. Sprenger A. Munte T. F. (2014). Changes in resting-state fMRI in vestibular neuritis. *Brain. Struct. Funct.* 219 1889–1900. 10.1007/s00429-013-0608-5 23881293

[B23] Kim C. H. Choi H. R. Choi S. Lee Y. S. Shin J. E. (2018). Patterns of nystagmus conversion in sudden sensorineural hearing loss with vertigo. *Medicine (Baltimore)* 97:e12982. 10.1097/md.0000000000012982 30412127 PMC6221715

[B24] Lanting C. P. de Kleine E. van Dijk P. (2009). Neural activity underlying tinnitus generation: results from PET and fMRI. *Hear Res.* 255 1–13. 10.1016/j.heares.2009.06.009 19545617

[B25] Li L. P. Shiao A. S. Chen L. F. Niddam D. M. Chang S. Y. Lien C. F. (2006). Healthy-side dominance of middle- and long-latency neuromagnetic fields in idiopathic sudden sensorineural hearing loss. *Eur. J. Neurosci.* 24 937–946. 10.1111/j.1460-9568.2006.04961.x 16930421

[B26] Liu Y. Wang K. Yu C. He Y. Zhou Y. Liang M. (2008). Regional homogeneity, functional connectivity and imaging markers of Alzheimer’s disease: a review of resting-state fMRI studies. *Neuropsychologia* 46 1648–1656. 10.1016/j.neuropsychologia.2008.01.027 18346763

[B27] Micarelli A. Chiaravalloti A. Viziano A. Danieli R. Schillaci O. Alessandrini M. (2017). Early cortical metabolic rearrangement related to clinical data in idiopathic sudden sensorineural hearing loss. *Hear Res.* 350 91–99. 10.1016/j.heares.2017.04.011 28460253

[B28] Michel O. (2011). The revised version of the german guidelines “sudden idiopathic sensorineural hearing loss”. *Laryngorhinootologie* 90 290–293. 21560090 10.1055/s-0031-1273721

[B29] Moore D. R. (1991). Anatomy and physiology of binaural hearing. *Audiology* 30 125–134. 10.3109/00206099109072878 1953442

[B30] Morita T. Hiraumi H. Fujiki N. Naito Y. Nagamine T. Fukuyama H. (2007). A recovery from enhancement of activation in auditory cortex of patients with idiopathic sudden sensorineural hearing loss. *Neurosci. Res.* 58 6–11. 17316856 10.1016/j.neures.2007.01.010

[B31] Moskowitz D. Lee K. J. Smith H. W. (1984). Steroid use in idiopathic sudden sensorineural hearing loss. *Laryngoscope* 94(5 Pt 1), 664–666. 10.1288/00005537-198405000-00016 6717224

[B32] Musiek F. Guenette L. Fitzgerald K. (2013). Lateralized auditory symptoms in central neuroaudiology disorder. *J. Am. Acad. Audiol.* 24 556–563. 10.3766/jaaa.24.7.4 24047943

[B33] Okuda T. Nagamachi S. Ushisako Y. Tono T. (2013). Glucose metabolism in the primary auditory cortex of postlingually deaf patients: an FDG-PET study. *ORL J. Otorhinolaryngol. Relat. Spec.* 75 342–349. 10.1159/000357474 24435067

[B34] Park H. M. Jung S. W. Rhee C. K. (2001). Vestibular diagnosis as prognostic indicator in sudden hearing loss with vertigo. *Acta Otolaryngol. Suppl.* 545 80–83. 10.1080/oto.121.533.80.83 11677749

[B35] Phillips D. P. Semple M. N. Calford M. B. Kitzes L. M. (1994). Level-dependent representation of stimulus frequency in cat primary auditory cortex. *Exp. Brain Res.* 102 210–226. 7705501 10.1007/BF00227510

[B36] Pogson J. M. Taylor R. L. Young A. S. McGarvie L. A. Flanagan S. Halmagyi G. M. (2016). Vertigo with sudden hearing loss: audio-vestibular characteristics. *J. Neurol.* 263, 2086–2096. 10.1007/s00415-016-8214-0 27435969

[B37] Power J. D. Barnes K. A. Snyder A. Z. Schlaggar B. L. Petersen S. E. (2012). Spurious but systematic correlations in functional connectivity MRI networks arise from subject motion. *Neuroimage* 59, 2142–2154. 10.1016/j.neuroimage.2011.10.018 22019881 PMC3254728

[B38] Power J. D. Barnes K. A. Snyder A. Z. Schlaggar B. L. Petersen S. E. (2013). Steps toward optimizing motion artifact removal in functional connectivity MRI; a reply to carp. *Neuroimage* 76, 439–441. 10.1016/j.neuroimage.2012.03.017 22440651 PMC3834590

[B39] Rauch S. D. (2008). Clinical practice. Idiopathic sudden sensorineural hearing loss. *N. Engl. J. Med.* 359 833–840. 18716300 10.1056/NEJMcp0802129

[B40] Rauch S. D. (2018). The clinical value of vertigo as a prognostic indicator of outcome in sudden sensorineural hearing loss. *JAMA Otolaryngol. Head Neck Surg.* 144 684–685. 10.1001/jamaoto.2018.0674 29931206

[B41] Shaia F. T. Sheehy J. L. (1976). Sudden sensori-neural hearing impairment: a report of 1,220 cases. *Laryngoscope* 86, 389–398. 10.1288/00005537-197603000-00008 1256213

[B42] Stachler R. J. Chandrasekhar S. S. Archer S. M. Rosenfeld R. M. Schwartz S. R. Barrs D. M. (2012). Clinical practice guideline: sudden hearing loss. *Otolaryngol. Head Neck Surg.* 146(3 Suppl) S1–S35. 22383545 10.1177/0194599812436449

[B43] Stephan T. Deutschländer A. Nolte A. Schneider E. Wiesmann M. Brandt T. (2005). Functional MRI of galvanic vestibular stimulation with alternating currents at different frequencies. *Neuro-Image* 26 721–732. 10.1016/j.neuroimage.2005.02.049 15955481

[B44] Wang C. T. Huang T. W. Kuo S. W. Cheng P. W. (2009). Correlation between audiovestibular function tests and hearing outcomes in severe to profound sudden sensorineural hearing loss. *Ear Hear* 30 110–114. 10.1097/aud.0b013e318192655e 19125033

[B45] Yan C. G. Craddock R. C. He Y. Milham M. P. (2013). Addressing head motion dependencies for small-world topologies in functional connectomics. *Front. Hum. Neurosci.* 7:910. 10.3389/fnhum.2013.00910 24421764 PMC3872728

[B46] Yao Z. Wang L. Lu Q. Liu H. Teng G. (2009). Regional homogeneity in depression and its relationship with separate depressive symptom clusters: a resting-state fMRI study. *J. Affect. Disord.* 115 430–438. 10.1016/j.jad.2008.10.013 19007997

[B47] Zhou F. Zhu M. C. Wang M. Wang H. T. Jiao Y. L. Huang L. F. (2018). Clinical analysis of idiopathic sudden sensorineural hearing loss with vertigo and without vertigo. *Lin Chuan Er Bi Yan Hou Tou Jing Wai Ke Za Zhi* 32 920–923. 10.13201/j.issn.1001-1781.2018.12.009 29921074

