# Supplementary figures and images for: Functional Magnetic Resonance Imaging Reveals Early Connectivity Changes in the Auditory and Vestibular Cortices in Idiopathic Sudden Sensorineural Hearing Loss With Vertigo: A Pilot Study
Source: Front Hum Neurosci. 2021 Sep 27;15:719254. doi: 10.3389/fnhum.2021.719254 (PMC8502874; doi:10.3389/fnhum.2021.719254)

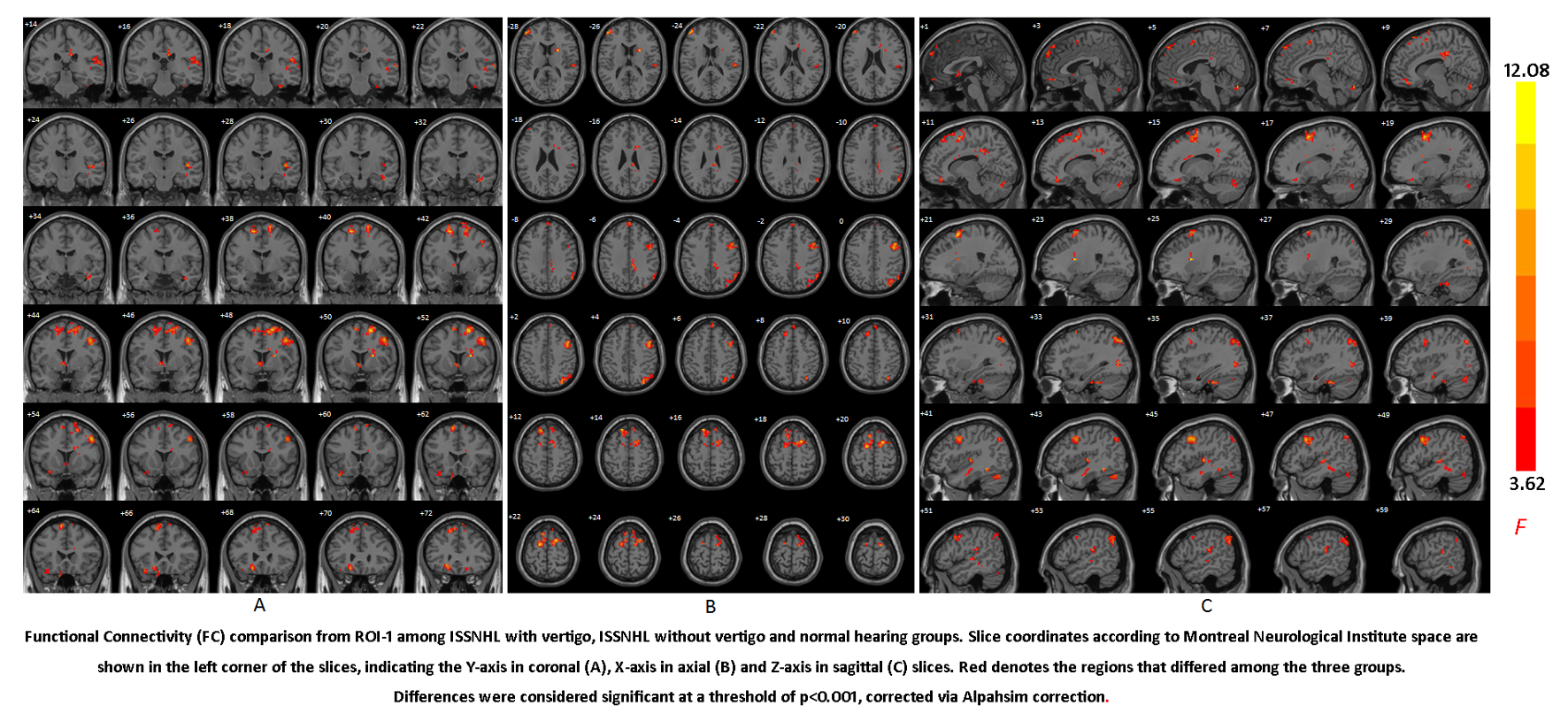

Supplement: Supplementary file 1 [file Image_1.TIF]

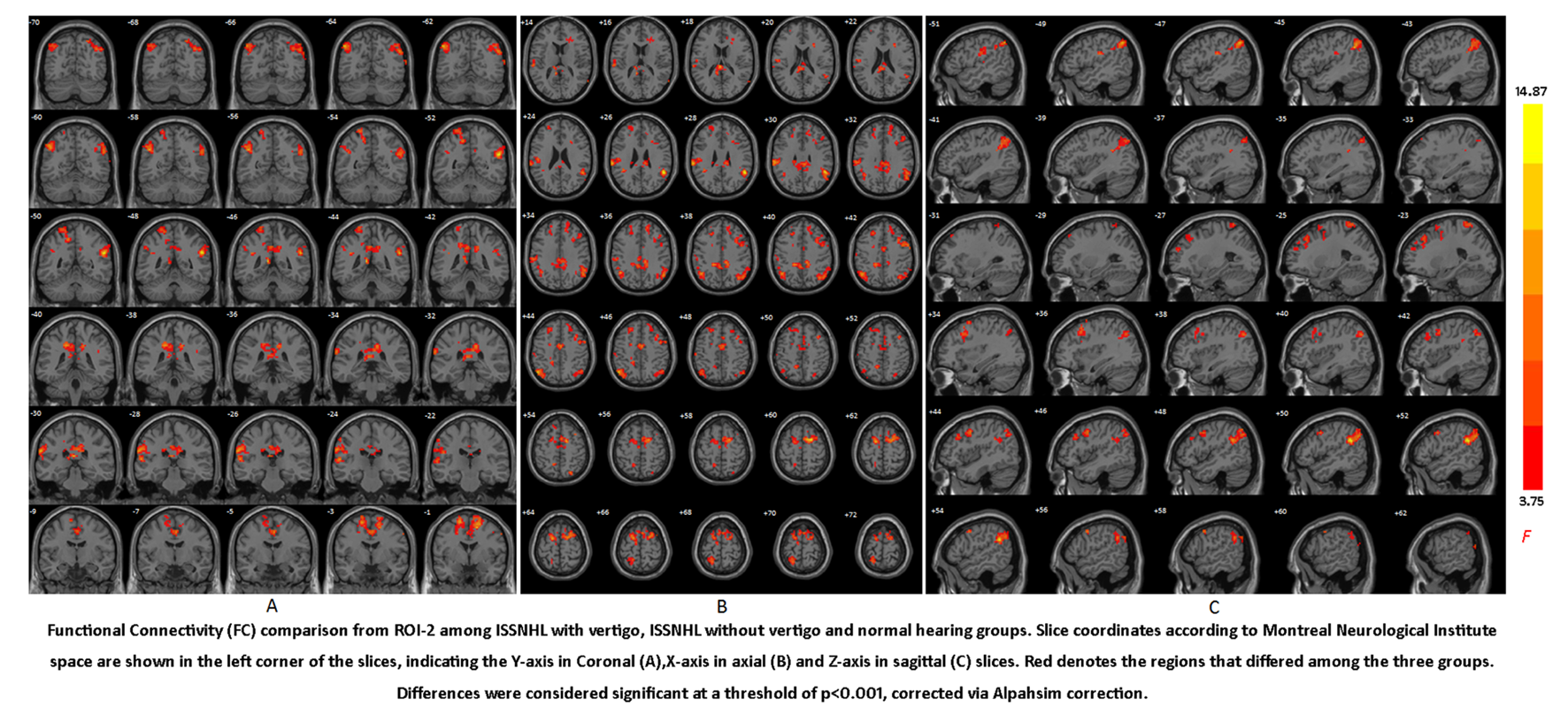

Supplement: Supplementary file 2 [file Image_2.TIF]
